# Nitrogen limitation and high density responses in rice suggest a role for ethylene under high density stress

**DOI:** 10.1186/1471-2164-15-681

**Published:** 2014-08-13

**Authors:** Maksym Misyura, David Guevara, Sanjeena Subedi, Darryl Hudson, Paul D McNicholas, Joseph Colasanti, Steven J Rothstein

**Affiliations:** Department of Molecular and Cellular Biology, University of Guelph, Guelph, ON Canada; Department of Mathematics & Statistics, University of Guelph, Guelph, ON Canada; Department of Mathematics and Statistics, McMaster University, Hamilton, Ontario Canada

**Keywords:** Rice, Nitrogen, Stress, Density, Competition, Microarray, Gene expression, Ethylene

## Abstract

**Background:**

High density stress, also known as intraspecies competition, causes significant yield losses in a wide variety of crop plants. At the same time, increases in density tolerance through selective breeding and the concomitant ability to plant crops at a higher population density has been one of the most important factors in the development of high yielding modern cultivars.

**Results:**

Physiological changes underlying high density stress were examined in *Oryza sativa* plants over the course of a life cycle by assessing differences in gene expression and metabolism. Moreover, the nitrogen limitation was examined in parallel with high density stress to gain a better understanding of physiological responses specific to high density stress. While both nitrogen limitation and high density resulted in decreased shoot fresh weight, tiller number, plant height and chlorophyll content, high density stress alone had a greater impact on physiological factors. Decreases in aspartate and glutamate concentration were found in plants grown under both stress conditions; however, high density stress had a more significant effect on the concentration of these amino acids. Global transcriptome analysis revealed a large proportion of genes with altered expression in response to both stresses. The presence of ethylene-associated genes in a majority of density responsive genes was investigated further. Expression of ethylene biosynthesis genes *ACC synthase 1*, *ACC synthase 2* and *ACC oxidase 7* were found to be upregulated in plants under high density stress. Plants at high density were also found to up regulate ethylene-associated genes and senescence genes, while cytokinin response and biosynthesis genes were down regulated, consistent with higher ethylene production.

**Conclusions:**

High density stress has similar but greater impact on plant growth and development compared to nitrogen limitation. Global transcriptome changes implicate ethylene as a volatile signal used to communicate proximity in under dense population growth condition and suggest a role for phytohormones in high density stress response in rice plants.

**Electronic supplementary material:**

The online version of this article (doi:10.1186/1471-2164-15-681) contains supplementary material, which is available to authorized users.

## Background

Recent estimates by the World Health Organization predict that the global population will approach 9 billion people by 2050 (http://www.who.int). Consequently, crop production will have to increase dramatically above current levels in order to sustain the human population. To increase yield per unit area one can either increase the amount an individual plant can produce or grow more plants per unit area. One of the challenges of the latter strategy is to maintain yield despite density stress imposed on crops planted in close proximity. Modern crop plants have been selected for their ability to produce higher yields per unit area, be more resistant to biotic and abiotic stresses, and have higher density tolerance [1–3]. Plant breeders have been selecting lines that better tolerate density stress, which has in turn led to a significant increase in the number of plants per unit area and thus increased yield [4]. However, the yield gains of major crops are predicted to decline in the near future, thus more innovative and targeted plant breeding strategies will be necessary to avoid food shortages in the future [5, 6]. Since crop productivity is adversely affected by a wide array of abiotic and biotic stressors, the elucidation of mechanisms underlying stress response in plants is important to ameliorate the negative effect of these factors on yield.

High density stress has been studied mostly from agronomic (e.g. yield) and physiological perspectives. Simple one-dimensional stresses, such as nitrogen limitation, have been studied in great detail over the years, while the more complicated/multi-factorial stresses, such as high density, have not received the same attention [7]. As planting density increases, yield exhibits a saturation-type curve and eventually plateaus; further increase in planting density beyond a certain point fails to increase yields and yield actually decreases when density gets too high [8]. Many years of artificial selection have produced crop plants that tolerate higher planting densities. For example, newer inbreds are able to grow better and maintain yields under high density stress compared to older ones due to more efficient resource capture [9]. Low plant-to-plant variability positively contributes to the yield increases in corn inbreds [9]. Furthermore, corn inbreds that were characterized as leafy with reduced stature were able to outperform other lines under high density growth conditions [10]. Modern varieties of osier willow (*Salix viminalis*) with more erect architecture were able to better tolerate high density stress [11]. However, selection of plant varieties with better high density tolerance does not address questions about mechanisms underlying high density stress response.

Molecular mechanisms underlying high density stress response have not been studied in great detail. High density stress has been typically examined in the context of shade avoidance response, especially as it pertains to changes in the ratio of red:far-red (R:FR) light [12]. However, high density stress also results in the shortage of nutrients, water, and light in addition to changes in light quality. The molecular and physiological response to the combination of these factors warrants further investigation of high density stress response under field like conditions. A recent microarray profiling study of barley and maize seedlings grown under high density stress found changes in genes associated with light intensity, auxin and lipid transfer were differentially expressed in barley and maize seedlings [13]. However, high population density stress did not result in major detectable changes in the transcriptome at the early stages of barley and maize life cycles despite detectable morphological changes [13]. In contrast, Arabidopsis studies have been able to identify groups of genes involved in high density stress response. For example, a significant portion of genes affected by high density stress are involved in nitrogen metabolism according to gene ontology classification [14]. Furthermore, brassinosteroid, salicylic acid analogue benzothiadiazole, abscisic acid and methyl jasmonate responsive genes were over-represented in plants grown under high population density condition [14]. Differential regulation of pathogen response genes has been observed in Arabidopsis plants grown under high density stress [15].

Various combinations of stresses and growth conditions have been used to identify relevant pathways underlying high density stress response. Intra-specific competition between rice plants and inter-specific competition between rice plants and weeds were used to determine general characteristics, such as upright leaf architecture and large leaf area, and infer similarities in genetic factors and physiological pathways between the two growth conditions [16]. Shading plants grown under various densities has been used to intensify the stress by increasing competition for light. Decreases in yield due to both stresses were attributed mainly to reduction in photosynthesis [17]. Drought can be one of many components of high density stress. A modern maize hybrid grown under high density stress with limiting amounts of water was found to up-regulate more genes in response to density stress according to global gene expression analysis of the roots. Furthermore, a greater proportion of differentially regulated genes in the newer hybrid were similar to drought response genes identified in a previous study by Seki and colleagues [18, 19].

The main objective of the current study was to utilize global gene expression and metabolite profiling to identify density specific responses in a model monocot. Limiting nitrogen (LN) was used in combination with high density (HD) stress to better delineate molecular pathways of high density stress response. The findings presented here describe changes in transcriptome and metabolome with a special focus on the potential role of ethylene. Our data support the notion that studies focused on stress combinations rather than individual stressors are able to provide novel information in the context of crop improvement.

## Results

### Plant growth response to high density and nitrogen limitation

**Figure 1:**
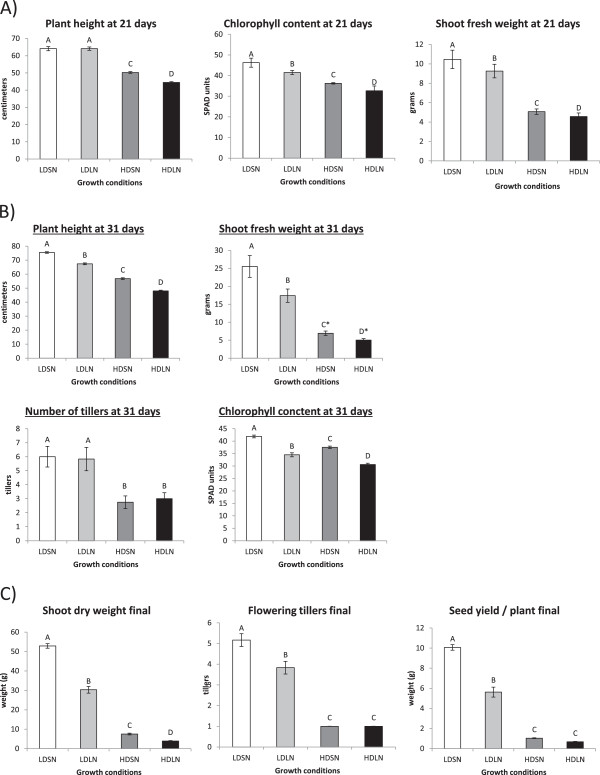
**Physiological and developmental characteristics of rice plants were negatively affected under high density and/or nitrogen (N) limitation conditions at 21 days (A) 31 days (B) and end of the life cycle (C).** All data is mean ± standard error with statistical significance determined using one-way ANOVA and the least significant difference at α = 5% (n = 5). No statistical significance according to Tukey (*).

The stress conditions used in this study (high density or HD, low density or LD, limiting nitrogen or LN, sufficient nitrogen or SN) allowed us to observe rice plants for a complete life cycle and set seed even under the least optimal (HDLN) growth condition. Rice plants grown under HD conditions had significantly fewer total (reproductive and vegetative) and reproductive tillers, were shorter, accumulated less shoot fresh weight and contained less chlorophyll compared to plants grown under low density growth conditions at all sampled time points and regardless of nitrogen levels (Figure 1A and B). HD and LN treatments had a long-term cumulative negative effect on plant growth and development throughout the life cycle of the plant. Specifically, differences in total tiller numbers, shoot fresh weight and plant height between plants grown under different density treatments at 31 days were greater compared to differences of the same parameters observed in 21 days old plants (Figure 1A and B). HD treatment resulted in 1.8 to 2.3-fold decrease in biomass at the 21 day time point and a 6.5-7.5 fold decrease in shoot dry weight at the end of the life cycle (Figure 1A). Nitrogen limitation resulted in little to no decrease in biomass at the 21 day time point and a 1.8 to 2-fold decrease in shoot dry weight at the end of the life cycle (Figure 1A). The decreases in shoot fresh and dry weight, plant height, seed yield, total and reproductive tiller number due to HD stress were greater than those caused by nitrogen limitation. HD stress and nitrogen limitation had varying effects on some aspects of plant growth and development. The response of plants to HD and limiting nitrogen treatment was similar with respect to most developmental and physiological characteristics, especially yield parameters such as biomass accumulation, plant height and seed production (Figure 1C). However, plants grown at HD condition produced the same number of tillers regardless of nitrogen supply at 31 days, as well as the same number of reproductive tillers at end of their life cycle (Figure 1C). In this case, the effect of HD stress superseded the effect of nitrogen limitation on plant growth and development. Therefore, it is important to note that some growth parameters responded differently to HD stress compared to limiting nitrogen condition.

#### Aspartate and glutamate levels vary under different growth conditions

**Figure 2:**
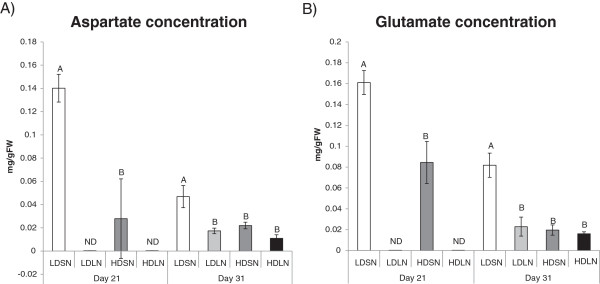
**Concentration of glutamate (A) and aspartate (B) in rice leaves was decreased due to high density and/or nitrogen limitation conditions sampled at 21 and 31 days according to GCMS analysis.** All data is mean ± 1 standard error with statistical significance determined using one-way ANOVA and the least significant difference at α = 5% (n = 3). Metabolites that were present at detectable levels and below quantifiable amount are displayed as “ND”.

Nitrogen limitation and HD stress are both associated with a reduction in biomass accumulation. Changes in aspartate and glutamate were correlated with decreases in biomass due to stress treatments. Metabolite profile changes of rice plants due to density stress resulted in decreases of glutamate and aspartate concentrations. Glutamate concentration is maintained at constant levels in a plant and only stress conditions, such as decrease in nitrogen nutrition, cause significant decrease in the glutamate concentration [20]. Therefore, glutamate measurements can be used to indicate relative nitrogen status of rice plants at various treatments and time points of the study. Aspartate metabolism is closely linked with exposure to stress and energy metabolism via the connection with the tricarboxylic acid cycle [21, 22]. Decreases in levels of both amino acids can be correlated with severity of stress conditions [21]. Therefore, accumulation of aspartate and glutamate can be used to assess overall physiological state of a plant. At 21 days, plants accumulated more glutamate at LDSN treatment compared to HDSN treatment (Figure 2). The difference in glutamate accumulation between these two treatments is nearly two fold. At 31 days, plants grown under LDSN conditions had approximately four times the glutamate concentration compared to other treatments (Figure 2). Change in glutamate concentration was much greater at 31 days, suggesting a more severe stress condition compared to 21 day time point. Furthermore, while glutamate and aspartate concentrations were affected by LN treatment at 21 days, there was no effect of LN treatment at HD growth condition at 31 days. Accumulation of aspartate was of particular interest to this study as it is a metabolic marker for biomass accumulation [23]. As noted by Sweetlove and colleagues [23], changes in the flux of the TCA cycle can be analyzed to determine whether plants are using more energy to increase biomass production or to maintain their current weight/size by expending energy on housekeeping processes. The shuttling of 2-oxoglutarate into production of amino acids, such as glutamate and aspartate, is one of the key indicators of biomass accumulation as opposed to ATP production [23]. At 21 days, LDSN grown plants had five times the aspartate concentration of plants grown under HD stress, which correlates with increased biomass accumulation (Figure 2). The pattern of both aspartate and glutamate accumulation changed over time. Differences in both aspartate and glutamate accumulation under LDSN conditions and other treatments involving one or both stressors were greater at 21 days than 31 days. Greater accumulation of both amino acids coincided with active growth stages, and preceded more significant differences in biomass accumulation observed at later time points. In addition, at the 31 day sampling time point there were no significant differences in accumulation of either amino acid in plants under HD conditions regardless of the nitrogen condition. Analogous to physiological measurements (Figure 1), growth under HD masked the effects of limiting nitrogen with respect to amino acid accumulation at 31 days (Figure 2). For the complete list of metabolites that were tested in this study see Additional file 1.

#### Transcriptome signatures of high density and nitrogen limitation stresses

**Figure 3:**
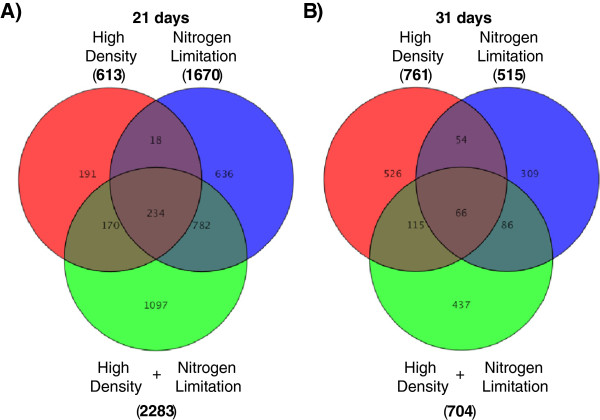
**Venn diagram showing overlap between transcripts responsive (enhanced or suppressed) to high density, nitrogen limitation and high density nitrogen limitation combined stress growth conditions.** The transcripts are sorted by time points: 21 days **(A)** and 31 days **(B)**, and have passed 2.0 fold cut-off with Benjamini-Hochberg FDR (p < 0.05).

To isolate genes that are differentially regulated in response to high density and not nitrogen limitation, transcriptome signatures of both high density and nitrogen limitation were compared while using the most optimal growth condition (LDSN) as the control. Furthermore, combined stress condition (HDLN) was included into the analysis to determine whether the transcriptome signature of combined stress is unique and is not simply the sum of HD and LN alone. The expression data, which was filtered based on 2.0 fold cut-off and Benjamini-Hochberg FDR (p > 0.05), revealed that 41% of genes responsive to high density stress were also responsive to nitrogen limitation at 21 days (Figure 3A). The proportion of genes responsive to high density stress and nitrogen limitation decreased to 16% at 31 days (Figure 3B). Combination of stresses at both times points produced transcriptome signatures that were unique from single stressors that made up this condition (Figure 3).

#### Cluster analysis of genes responsive to density stress identified co-regulated genes

**Figure 4:**
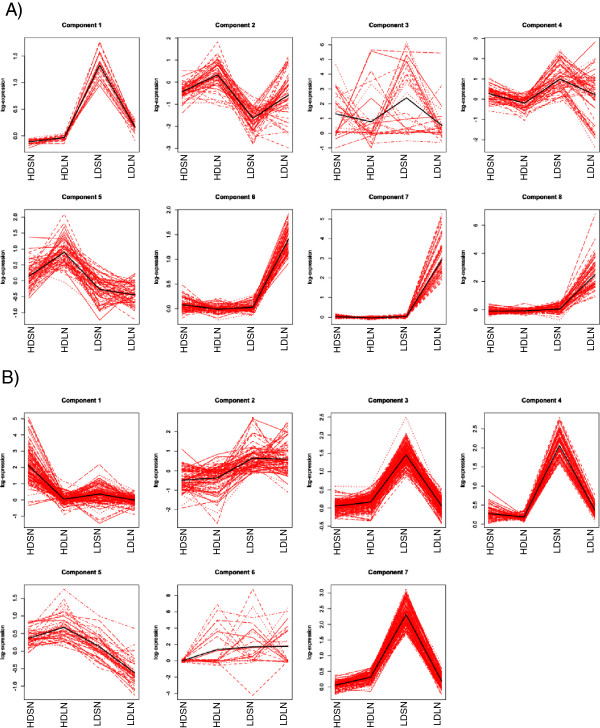
**Cluster analysis of differentially expressed genes at 21 (A) and 31 (B) days in rice plants grown under varying density and nitrogen treatments.** The vertical axis corresponds to the normalized expression levels in the log-scale, the horizontal axis corresponds to the treatments: low density (LD), high density(HD), limiting nitrogen(LN), sufficient nitrogen(SN). Black line represents the respective means.

To gain better insight into the physiology of high density stress, differentially expressed genes associated with high density stress and not nitrogen limitation were analyzed from this point forward. Cluster analysis was performed to identify gene groups with potentially similar functions. Out of 21,179 genes, 426 genes showed differential expression at 21 days and 707 genes showed differential expression at 31 days due to HD stress treatment using three-fold cut-off. Mixture model-based clustering was performed using multivariate Gaussian distributions with an eigen-decomposed covariance structure (Figure 4) [24, 25]. The data were also analyzed using parsimonious Gaussian mixture models [26]. Genes that passed the cut-off were sorted into groups based on expression profiles at various growth conditions. Eight clusters at 21 days and seven clusters at 31 days were chosen based on Bayesian information criterion (Figure 3). The average expression pattern for groups of genes (represented by the black lines in Figure 3) in each cluster was used to determine the response of these genes to various growth conditions. Groups of genes responsive to only high density, nitrogen limitation or both stresses were evident using this approach (see Additional file 2 for gene lists of individual clusters) further supporting the argument that high density stress response produces a unique transcriptome signature.

#### Genes associated with ethylene status are differentially expressed in rice plants grown under high density stress

We used microarray data and MapMan analysis to assess potential functions of genes responsive to HD stress and not LN. The processes and pathways responsive to HD stress were determined using MapMan analysis (Additional file 3). A wide variety of genes were differentially expressed in rice plants grown at high density (for example see Additional file 4 for AgriGO summary analysis of HD responsive genes at 31 days). Changes in the expression of a small number of each of the phytohormone metabolism and signal transduction pathway genes of abscisic acid, auxin, brassinosteroids, cytokinin, gibberellin and jasmonate genes were found by cluster analysis and pairwise comparison according to MapMan classification (Additional file 4). However, in the case of the ethylene-associated genes a relatively large number of the genes involved in biosynthesis, signal transduction and response had modified levels of expression of at least one member of the corresponding gene family. Therefore, ethylene metabolism and ethylene responsive genes were investigated further.

**Figure 5:**
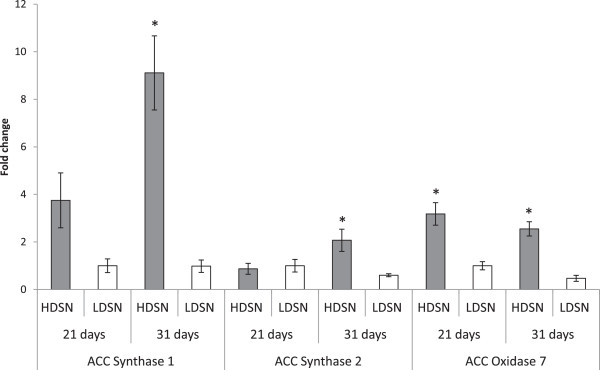
**Expression levels of ethylene biosynthesis genes** ***ACC synthase 1*** **,** ***ACC synthase 2*** **and** ***ACC oxidase 7*** **of rice plants grown under high density (HD) and low density (LD) at 21 and 31 days.** All data is mean ± SEM. For statistical significance (*) *t*-test was applied for every pair of means for a given gene at a given time point (p < 0.05, n = 3).

Ethylene modulates various aspects of plant stress response in plants [27]. Expression of ethylene synthesis and response genes was investigated further to determine the state of ethylene metabolism in response to HD. The changes in gene expression of three ethylene biosynthesis genes were confirmed by qPCR to determine the state of ethylene metabolism in response to HD (Figure 4). Previous studies have used ethylene production genes expression, such as *ACC oxidase* and *ACC synthase*, as a proxy to measure ethylene hormone production [28, 29]. Expression analysis of ethylene response genes was conducted to support the argument that HD stress modulates ethylene production in rice plants. qPCR analysis verified that ethylene biosynthesis genes were altered by HD stress. The expression of *ACC synthase* genes *(ACS) 1*, *ACS2* and ACC oxidase 7 (*ACO7)* increased over time in plants subjected to HD stress confirming microarray prediction of elevated ethylene production levels under HD stress (Figure 5). At 31 days under HD conditions the expression level of *ACS1* was nine times higher and that of *ACS2* was over 3 times higher HD compared to plants grown under low-density conditions. No statistically significant differences were detected in expression levels of *ACS1* and *ACS2* genes at 21 days at various density treatments. *ACO7* expression was threefold higher in plants grown under HD treatment at 21 days and fivefold higher at 31 days.

#### Ethylene response associated genes are differentially regulated in rice plants grown under high density stress treatment

**Figure 6:**
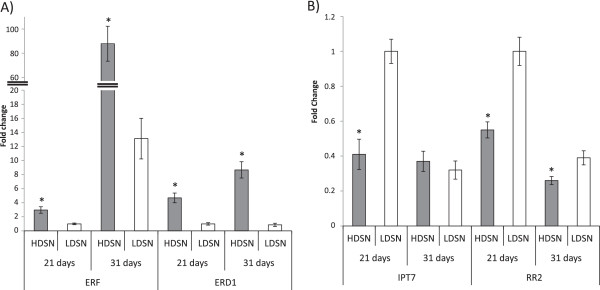
**Expression analysis of ethylene associated genes in plants grown under high density stress.** Ethylene responsive genes **(A)** and ethylene antagonist genes belonging to cytokinin production and response **(B)** demonstrated opposite patterns of expression under high density stress. All data is mean ± 1 standard error. For statistical significance (*) *t*-test was applied for every pair of means for a given gene at a given time point (p < 0.05, n = 3).

Ethylene has number of downstream targets, such as *ETHYLENE RESPONSE FACTOR* (*ERF*) and *EARLY RESPONSE TO DROUGHT 1* (*ERD1*) genes [30, 31]. Expression of cytokinin production and response genes, as well as ethylene response genes were all found to be affected by HD stress (Figure 6). Cytokinin is a well-known antagonist of ethylene in plants and as such was expected to show reduced biosynthesis and/or response under HD stress [32]. Expression levels of a predicted *ERF* and *ERD1* genes were elevated in HD grown rice plants in our experiment, further confirming increased ethylene production (Figure 6A). Genes that contain an ERF domain have been shown to respond to ethylene and are involved in ethylene responsive gene transcription [33]. The levels of *ERF* and *ERD1* transcript were consistently higher in HD grown plants at both time points (Figure 6A). The expression of *ERF* in HD plants was threefold higher at 21 day and 6.6-fold higher at the 31 day time point. Expression of *ERD1* was 4.7-fold higher at 21 days and 10-fold higher at 31 day time point. It should be noted that the *ERD1* gene serves double role because it is has been shown to respond to ethylene and is part of the senescence process [34].

Expression of the cytokinin biosynthesis gene *ISOPENTYL TRANSFERASE 7* (*IPT7)* and *RESPONSE REGULATOR* gene (*RR2)* were 2.4 and 1.81-fold down-regulated in plants grown under HD versus low density growth condition at 21 days (Figure 6B). Expression of *IPT7* was not significantly different at 31 days in plants grown under LD and HD conditions. While small differences (~1.5 fold) in the expression level of *RR2* were detected at 31 days, it is unlikely to correspond with a physiological difference (Figure 6B). Therefore, this suggests that plants were undergoing more active growth at the 21 day time point under low density growth conditions. In addition, ethylene homeostasis is likely differentially regulated in plants grown under HD conditions as indicated by changes in the expression of genes associated with ethylene production and response, as well as cytokinin biosynthesis and response.

#### High density grown plants express higher levels of senescence-associated genes at 21 days

**Figure 7:**
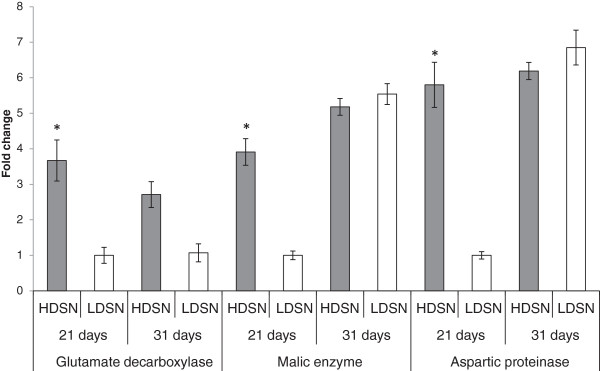
**Expression of three senescence associated genes in plants grown under high (HD) and low (LD) density conditions at both 21 and 31 day time points.** All data is mean ± 1 standard error. For statistical significance (*) *t*-test was applied for every pair of means for a given gene at a given time point (p < 0.05, n = 3).

Plants grown under HD stress showed downstream effects supporting changes in ethylene homeostasis, such as decreased growth and accelerated senescence. Since ethylene is known to be involved in senescence, senescence associated genes were expected to have different expression patterns in plants under HD stress [35]. Several senescence associated genes, including glutamate decarboxylase, malic enzyme and aspartic proteinase, were selected for analysis based on previously published results to further confirm changes in ethylene homeostasis [36]. The expression of glutamate decarboxylase was 3.7 fold higher in plants grown under HD stress at 21, while no statistically significant difference was found at 31 days. Malic enzyme and aspartic proteinase encoding genes were found to have 3.9 and 5.8-fold higher expression in HD grown plants only at the 21 day time point (Figure 7).

## Discussion

Global transcriptome and metabolic changes of rice grown under HD and/or LN growth conditions were examined to investigate major pathways involved in high population density stress response in rice. While previous studies examined the response of crop plants to HD stress with respect to yield and therefore assessed the final resulting outcome, the current study aimed to identify molecular mechanisms of HD stress response in rice during the life cycle of a plant [37, 38]. The negative impact of HD stress on plants is well known, but limited research has been done to determine whether high population density affects plants at the molecular and genetic levels. Moreover, the mechanisms underlying plant stress response, with the exception of shade avoidance, have not been examined in great detail in the context of HD stress. Furthermore, since HD stress is composed of a number of factors nitrogen limitation was examined in parallel.

### High density and limiting nitrogen have similar but varying impacts on growth parameters, transcriptome and metabolism

Plant growth characteristics, such as height, chlorophyll content, weight and tiller number, decreased in response to both HD and/or LN growth conditions. Both stressors also had varying impacts on growth characteristics (Figure 1). While nitrogenn supply and the number of plants grown per unit area are known to be correlated with a number of plant growth parameters, there were significant differences in the impact of HD and LN treatments on plant growth. Previous research has shown the positive effect of ply and density on various aspects of plant growth and development [39, 40]. Since the negative effects on plant growth of LN and HD are known, this study focused on determining physiological responses that are unique to high density stress. Under the conditions used in our study, the reduced height of rice plants grown under HD stress was apparent in 21 days old plants and persisted throughout the life cycle. Increase in plant height in response to increasing density has been shown to follow a saturation curve with density not having an effect on plant height above a certain threshold [41]. Our findings demonstrate that reduction in plant height observed under HD treatment was likely due to the reduction in available resources required for plant growth, such as nitrogen.

### Aspartate and glutamate concentration are decreased under high density and nitrogen limitation

Metabolite analysis revealed similar physiological responses triggered by both LN and HD stress. Decreases in accumulation of aspartate and glutamate were observed under HD stress and LN (Figure 2). Nitrogen is known to be one of the most important factors for plant growth [42]. Levels of various resources, such as nutrients, physical growth space and light, are all limited by the increase in the number of plants per unit area under the HD treatment. Furthermore, nitrogen supply is directly linked to glutamate and aspartate accumulation [21]. However, nitrogen alone does not necessarily explain the drastic reduction in growth under HD stress conditions. Stress combinations can produce results that are different than the additive effects of individual stressors. For example, the combination of heat and drought elicits unique responses in plants that are not observed under individual treatments [43].

### Comparison of trancriptome signatures under high density and nitrogen limitation stress

Comparison of global transcriptome changes due to HD and limiting nitrogen stresses revealed a large number of the same genes responsive to both treatments (Figure 3). In a recent study, genes related to nitrogen metabolism were demonstrated to be enriched among HD stress responsive genes [14]. Therefore, our results in rice agree with previous findings in Arabidopsis (Additional file 4). However, the similarity between changes in global transcriptome induced by HD and nitrogen limitation decreased over time (Figure 3). According to our results, global transcriptome changes due to HD stress are significantly different from LN at later stages in plant growth and development despite the significant contribution of nitrogen limitation to the observed phenotypic changes.

### Unique physiological consequences of high density stress response

Density stress is known to activate genes that are not involved in nutrient deficiency or abiotic stress, such as herbivory, and bacterial and fungal infection response genes [14]. Biotic stress is a common functional category of genes induced by HD. Biotic stress was common functional category of genes induced by HD stress in both Arabidopsis and *Solanum nigrum* [14, 44]. MapMan analysis identified a number of genes similar to Arabidopsis genes involved in biotic stress response, such as *RPM1* (AT3G07040), *NB-ARC* (AT3G14470) and *ATHCHIB* (AT3G12500), among differentially expressed genes due to HD stress. However, biotic stress response-associated genes were not highly enriched in our study according to AgriGO analysis (Additional file 4) [45]. Therefore our results stand in contrast to previous findings in Arabidopsis where pathogen defense pathways were strongly down-regulated in response to HD stress [15].

Our findings suggest that rice plants utilize ethylene to communicate their proximity to neighbouring plants in a high population density environment. Expression differences in ethylene-associated genes have been detected in high population density studies in *Arabidopsis* and *Solanum nigrum* [14, 44]. The major phytohormone-responsive gene categories induced by high population density stress in *Arabidopsis* were related to benzothiadiazole (a salicylic acid analogue), abscisic acid and methyl jasmonate, while ethylene represented a lesser proportion of differentially regulated genes [14]. Only a single ethylene receptor homologue in *Solanum nigrum* was identified to be responsive to HD stress [44]. In contrast, here we find that a relatively large number of ethylene related genes are differentially expressed compared to other phytohormones in rice plants due to HD stress. Therefore ethylene might play a role in HD stress response in rice and serve as a plant-plant communication signal. Expression analysis of well-known ethylene associated genes confirmed changes in ethylene homeostasis, further implicating the phytohormone in HD stress response. A number of studies in tobacco and *Arabidopsis* demonstrated that ethylene is involved in shade avoidance response [46–49]. In tobacco, ethylene-insensitive mutant plants were out-competed by the wild type neighbors due to inability of the mutants to sense changes in blue light [48, 49]. On the other hand, ethylene was implicated in sensing changes in the R:FR ratio in Arabidopsis plants [46]. However, research centered on the role of ethylene in HD stress response of monocots, especially agronomically important plants such as rice, has been lacking. Our results support previous findings in model dicots, as well as suggest a role for ethylene in shade avoidance response in rice and possibly other monocots.

Changes in light quality, characterized mainly by changes in R:FR ratio, ethylene production and touch sensitivity mechanisms might all contribute to the shade avoidance response in rice under HD stress. The contribution of ethylene to shade avoidance response was initially underestimated [49]. While ethylene does not take part in R:FR mediated hyponasty, the hormone is required for both blue light and ethylene mediated shade avoidance response [49]. The importance of ethylene in plant response to external stimuli as the driving force behind growth and developmental changes are now well known [50]. Ethylene production could be a major factor in neighbor recognition in rice plants under HD stress in conjunction with R:FR ratio and blue light perception machinery. The upright architecture of rice plants minimizes the amount of shade imposed on neighbouring plants compared to *Arabidopsis* and tobacco plants. Lastly, a recent study in *Arabidopsis* demonstrated that touching of leaves precedes R:FR driven phytochrome signaling [51]. Thus the hyponasty of rice plants may be driven primarily by ethylene and touch-related pathways during the initial stages of growth.

## Conclusions

The molecular basis of HD stress response has not been studied extensively. The main factor for this is mostly likely the multitude of factors involved in HD stress, such as various resource limitations. A multiple stress combination approach could be necessary to decipher all of the pathways involved in HD stress response. Similarities between limiting nitrogen and HD stress response were found with respect to a number of developmental and physiological characteristics, such as biomass accumulation, decrease in tiller number, chlorophyll, and aspartate and glutamate concentration. Furthermore, HD stress resulted in global transcriptome changes similar to those elicited by nitrogen limitation at the early stages of plant growth and development. Changes in ethylene homeostasis in response to HD stress implicate ethylene as a plant-plant communication signal under HD stress. While the molecular mechanisms of ethylene driven shade avoidance response are beginning to emerge, future studies in crop plants remain to be conducted [46, 52]. Ethylene-related pathways may be viable targets for breeding crop plants that are more tolerant to HD stress. The research presented here demonstrates the importance of multi-dimensional approaches to deciphering density stress responses and highlights the need for further investigations into the impact of combinatorial stresses on plant development and yield.

## Methods

### Experimental design and rationale

Rice plants were grown under four different conditions: high density and sufficient nitrogen (HDSN), high density and limiting nitrogen (HDLN), low density and sufficient nitrogen (LDSN), low density and limiting nitrogen (LDLN). Low density condition (6 plants per bin with 20cm spacing between individual plants) was chosen to resemble rice density under normal field conditions (Additional file 3) [53]. High density condition (40 plants per bin with 8 cm spacing between individual plants) was chosen to maximize the effects of high density stress while allowing plants to complete their life cycle (Additional file 3: Figure S1). Nitrogen was supplied in pre-determined quantities as well. Sufficient nitrogen (SN) condition was defined as 10mM NO_3_^-^ and limiting nitrogen (LN) condition was set at 3mM NO_3_^-^ [54]. Physiological measurements were taken to assess the relative impact of high density and nitrogen limitation on growth and development of rice plants. Sampling times were selected based on phenotypic characteristics, such as plant size and height. At 21 days rice plants were undergoing active period of growth and the differences in various growth growth parameters were observed between various treatments (Figure 1). At 31 days rice plants were not exhibiting signs of active growth and were induced to flower by administering a short day treatment for one day. Since the plants senesced at different rates, it was no longer applicable to measure shoot fresh weight at a single time point. Instead, dry weight was assessed after all of the rice plants from all of the treatments had dried out.

#### Plant growth conditions

The plants were grown in Rubbermaid containers (61 × 41 × 32 cm) at 450 μmol m^-2^ s^-1^, 16 h day at 27°C/8 h night at 23°C and 75% relative humidity. High density treatment was defined as 40 plants per container and low density treatment was defined as 6 plants per container, uniformly spaced. The samples for gene expression and metabolite analysis were collected from 21 and 31 days old plants. The leaf tissues were frozen immediately in liquid nitrogen.

#### Gene expression: microarray and qRT-PCR

Total RNA was isolated from 100mg of frozen leaf tissue with TRIzol reagent (Invitrogen). RNeasy (Qiagen) columns were used to purify the RNA samples. 1 mg of total RNA was converted into cDNA using Superscript (Quanta) kit. Three biological replicates were used for microarray analysis.

For global expression analysis, Rice Genome OneArray® Microarray chips were used. Labelling, hybridization, and scanning of the chips were carried out by the University of Guelph Genomics facility (Guelph, Ontario, Canada) according to the manufacturer’s protocols (http://www.phalanxbiotech.com). The .cel files were loaded into GeneSpring GX 11 (Agilent Technologies) and the GC-RMA normalization algorithm was applied. A minimum 2-fold change between normalized averages were used as a cut-off point setting for further analysis and Benjamini-Hochberg FDR was applied (p < 0.05). MapMan pathway analysis was used to assign differentially expressed genes to functional bins [55]. AgriGO analysis was carried out on differentially expressed genes to determine enriched gene ontology terms [45].

Relative quantification was achieved by comparing expression of genes of interest to *actin2* control. Three biological replicates at HDSN were compared to LDSN. Reactions were performed on 8-fold diluted samples of cDNA and incubated at 50°C for 2 min and 95°C for 2 min, cycled at 95°C for 15 s, and 60°C for 60 s for 40 cycles with PerfeCTa SYBR Green SuperMix (Quanta Biosciences). Applied Biosystems 7300 Real Time PCR instrumentwas used for data collection, and the resulting data were analysed by the 2^–∆∆Ct^ method to obtain fold difference in expression between genes of interest compared to optimal (LDSN) condition [56]. See Additional file 5 for all of the primers used in the study.

#### Metabolic profiling

The polar fraction was lyophilized and derivatized using methoxyamine and *N*-methyl-*N*-trimethylsilyl-trifluoroacetamide as previously described [57]. A 1 μl aliquot of derivatized sample was injected into the splitless injection port of a Varian 1200 GC-MS system (Varian). Chromatography was performed using an Rtx-5MS column (Chromatographic Specialties, Brockville, Ontario, Canada).

Data analysis was performed using the automated mass spectral deconvolution and identification system (AMDIS, http://chemdata.nist.gov/mass-spc/amdis). The resulting components were filtered in GASP [58] using a signal to noise ratio of 5 as a cut-off requirement. Component data were normalized to ribitol, and average normalized component area was compared between samples. The Golm Metabolite Database was used for component identification [59].

#### Statistical analysis

For data where only 2 means were compared (2 growth conditions for a single biomarker at a given time point), students *t*-test was applied (p < 0.05). For multiple mean comparison, statistical significance was determined using Statistix 9.0 software program. The mean values were analyzed using one-way ANOVA and the least significant difference at α = 5% was recorded. Furthermore, Tukey post-hoc test was applied and a note was made where the samples did not pass p < 0.05 threshold. To obtain normal distribution according to Shapiro-Wilk test, data were square-root transformed.

#### Cluster analysis

Using the measurements of all 4 conditions, the genes were clustered via a model‒based clustering approach for each time point separately. Model‒based clustering assumes that the population is a convex combination of probability densities; i.e., that a mixture of subpopulations each with its own mean and covariance structure. In the analysis, Gaussian parsimonious clustering models [25]) were used, a subset of which is implemented in the R package mclust [26, 60]. This family of model utilizes eigen‒decomposed component covariance structures.

A two-step clustering strategy was used for the analysis of each time point (for full explanation of cluster analysis see Additional file 6 “Cluster analysis”).

Step 1 In this step, using the measurements on all 4 conditions, the genes were clustered via a model-based clustering approach for each time point separately. This step ensures that genes that follow a similar trend are clustered together. In this step, a subset of GPCM available in the R package mclust were used.

Step 2 In the second step, the measurements on the four conditions from each component were split according to the plant density. This resulted in a two dimensional data such that the first set contained expression levels under high and limiting nitrogen, and high density and the second set contained expression levels under high and limiting nitrogen, and low density. Each component was analyzed using k-means with k = 2. k-means approach partitions data into k clusters such that each observation belongs to the cluster with the nearest mean. If a gene’s response to nitrogen follows a particular trend under low density but follows a different trend under high density, the resulting two clusters will represent such trends.

The data was also analyzed using other clustering approaches.

1. Parsimonious Gaussian mixture models [61] for step 1 and k-means with k = 2 for step 2. PGMM utilizes a latent factor structure for the data and is available in the R package pgmm [62].
2. GPCM for step 1 followed by GPCM with G = 2 for step 2.
3. PGMM for step 1 followed by GPCM with G = 2 for step 2.

### Supporting data

The data for microarray experiment was submitted to NCBI GEO public repository and can be found at http://www.ncbi.nlm.nih.gov/geo/query/acc.cgi?acc=GSE57094.

## Electronic supplementary material

Additional file 1: **Summary of all metabolites analyzed by GC-MS at both 21 and 31 days.** (DOCX 26 KB)

Additional file 2: **Gene lists identified by the cluster analysis.** (XLSX 24 KB)

Additional file 3: **Growth conditions used in this study are shown in** **Figure S1. Figure S2.** shows an example of MapMan analysis output and demonstrates involvement of ethylene hormone. (DOCX 2 MB)

Additional file 4: **Summary of AgriGO analysis of differentially expressed genes at 31 days.** (DOCX 21 KB)

Additional file 5: **Sequences of primer pairs that were used to analyze gene expression by qRT-PCR.** (XLSX 11 KB)

Additional file 6: **Detailed description of cluster analysis.** (DOCX 943 KB)
