# Supplementary material for: Nitrogen limitation and high density responses in rice suggest a role for ethylene under high density stress
Source: BMC Genomics. 2014 Aug 13;15(1):681. doi: 10.1186/1471-2164-15-681 (PMC4138374; doi:10.1186/1471-2164-15-681)
Supplement: Supplementary file 1 — Additional file 1: Summary of all metabolites analyzed by GC-MS at both 21 and 31 days. (DOCX 26 KB) [file 12864_2013_6360_MOESM1_ESM.docx]

**Additional file 1**

Table S1 Summary of metabolites analyzed by GCMS at 21 days.

|  | **High Density** | | | | | | **Low Density** | | | | | |
| --- | --- | --- | --- | --- | --- | --- | --- | --- | --- | --- | --- | --- |
|  | **High N** | | | **Low N** | | | **High N** | | | **Low N** | | |
| **Alanine** | 3.00E-04 | ± | 0.00E+00 | 3.00E-04 | ± | 0.00E+00 | 3.00E-04 | ± | 0.00E+00 | 3.00E-04 | ± | 0.00E+00 |
| **Citrate** | 5.34E-02 | ± | 1.24E-02 | 3.25E-02 | ± | 5.58E-02 | 6.26E-02 | ± | 3.03E-02 | 3.00E-04 | ± | 0.00E+00 |
| **Ethanolamine** | 3.00E-04 | ± | 0.00E+00 | 3.00E-04 | ± | 0.00E+00 | 3.00E-04 | ± | 0.00E+00 | 3.00E-04 | ± | 0.00E+00 |
| **Fructose MeOX1** | 6.78E-01 | ± | 2.22E-01 | 7.75E-01 | ± | 8.62E-01 | 1.21E+00 | ± | 2.79E-01 | 1.14E+00 | ± | 3.36E-01 |
| **Fructose MeOX2** | 4.36E-01 | ± | 1.62E-01 | 5.09E-01 | ± | 5.67E-01 | 7.22E-01 | ± | 1.72E-01 | 6.15E-01 | ± | 1.66E-01 |
| **Galactose MeOX1** | 3.00E-04 | ± | 0.00E+00 | 3.00E-04 | ± | 0.00E+00 | 3.00E-04 | ± | 0.00E+00 | 3.00E-04 | ± | 0.00E+00 |
| **Glucose MeOX1** | 7.51E-01 | ± | 2.66E-01 | 1.01E+00 | ± | 1.23E+00 | 1.36E+00 | ± | 3.00E-01 | 1.28E+00 | ± | 4.12E-01 |
| **Glucose MeOX2** | 6.62E-02 | ± | 2.07E-02 | 8.13E-02 | ± | 1.06E-01 | 1.72E-01 | ± | 8.01E-02 | 1.45E-01 | ± | 3.32E-02 |
| **Glycerol** | 3.00E-04 | ± | 0.00E+00 | 3.00E-04 | ± | 0.00E+00 | 3.00E-04 | ± | 0.00E+00 | 3.00E-04 | ± | 0.00E+00 |
| **Glycine** | 3.00E-04 | ± | 0.00E+00 | 3.00E-04 | ± | 0.00E+00 | 3.00E-04 | ± | 0.00E+00 | 3.00E-04 | ± | 0.00E+00 |
| **Isocitrate** | 3.00E-04 | ± | 0.00E+00 | 3.00E-04 | ± | 0.00E+00 | 3.00E-04 | ± | 0.00E+00 | 3.00E-04 | ± | 0.00E+00 |
| **Leucine** | 3.00E-04 | ± | 0.00E+00 | 3.00E-04 | ± | 0.00E+00 | 3.00E-04 | ± | 0.00E+00 | 3.00E-04 | ± | 0.00E+00 |
| **Malate** | 8.04E-01 | ± | 5.28E-01 | 3.00E-04 | ± | 0.00E+00 | 7.89E-01 | ± | 2.95E-01 | 7.19E-01 | ± | 7.46E-01 |
| **Myo-inositol** | 1.39E-01 | ± | 2.23E-02 | 2.76E-01 | ± | 2.56E-01 | 1.61E-01 | ± | 1.06E-01 | 1.06E-01 | ± | 4.75E-02 |
| **Phosphate** | 3.00E-04 | ± | 0.00E+00 | 3.00E-04 | ± | 0.00E+00 | 3.16E-01 | ± | 1.25E-01 | 3.00E-04 | ± | 0.00E+00 |
| **Pyroglutamate** | 1.01E-02 | ± | 3.53E-03 | 3.00E-04 | ± | 0.00E+00 | 1.98E-01 | ± | 3.73E-02 | 3.00E-04 | ± | 0.00E+00 |
| **Quinate** | 3.13E-01 | ± | 1.65E-02 | 5.01E-01 | ± | 2.16E-01 | 3.78E-01 | ± | 1.85E-01 | 5.59E-01 | ± | 5.38E-02 |
| **Serine** | 3.00E-04 | ± | 0.00E+00 | 3.00E-04 | ± | 0.00E+00 | 1.45E-01 | ± | 6.32E-02 | 3.00E-04 | ± | 0.00E+00 |
| **Succinate** | 3.00E-04 | ± | 0.00E+00 | 3.00E-04 | ± | 0.00E+00 | 2.62E-02 | ± | 2.09E-02 | 3.00E-04 | ± | 0.00E+00 |
| **Sucrose** | 6.60E+00 | ± | 2.31E+00 | 2.23E+01 | ± | 2.70E+01 | 7.32E+00 | ± | 1.99E+00 | 2.14E+01 | ± | 2.51E+01 |
| **Threonate** | 1.14E-02 | ± | 1.15E-02 | 3.00E-04 | ± | 0.00E+00 | 3.04E-02 | ± | 1.52E-02 | 3.00E-04 | ± | 0.00E+00 |
| **Threonine** | 3.00E-04 | ± | 0.00E+00 | 3.00E-04 | ± | 0.00E+00 | 6.33E-02 | ± | 2.89E-02 | 3.00E-04 | ± | 0.00E+00 |
| **Valine** | 3.00E-04 | ± | 0.00E+00 | 3.00E-04 | ± | 0.00E+00 | 3.00E-04 | ± | 0.00E+00 | 3.00E-04 | ± | 0.00E+00 |

Table S2 Summary of metabolites analyzed by GCMS at 31 days

|  | **High Density** | | | | | | **Low Density** | | | | | |
| --- | --- | --- | --- | --- | --- | --- | --- | --- | --- | --- | --- | --- |
|  | **High N** | | | **Low N** | | | **High N** | | | **Low N** | | |
| **Alanine** | 3.00E-04 | ± | 0.00E+00 | 1.16E-02 | ± | 1.08E-02 | 4.14E-02 | ± | 7.19E-03 | 2.01E-02 | ± | 3.64E-03 |
| **Citrate** | 5.53E-02 | ± | 5.36E-02 | 6.86E-02 | ± | 4.99E-02 | 1.31E-01 | ± | 4.51E-02 | 1.13E-01 | ± | 2.62E-02 |
| **Ethanolamine** | 3.00E-04 | ± | 0.00E+00 | 3.00E-04 | ± | 0.00E+00 | 7.02E-03 | ± | 2.33E-03 | 4.96E-03 | ± | 3.14E-03 |
| **Fructose MeOX1** | 1.11E+00 | ± | 1.70E-01 | 6.23E-01 | ± | 1.57E-01 | 2.53E+00 | ± | 5.67E-01 | 1.29E+00 | ± | 3.37E-01 |
| **Fructose MeOX2** | 6.97E-01 | ± | 7.42E-02 | 3.68E-01 | ± | 1.02E-01 | 1.20E+00 | ± | 4.71E-01 | 8.12E-01 | ± | 1.98E-01 |
| **Galactose MeOX1** | 3.00E-04 | ± | 0.00E+00 | 3.00E-04 | ± | 0.00E+00 | 3.65E-03 | ± | 1.41E-03 | 2.68E-03 | ± | 2.20E-03 |
| **Glucose MeOX1** | 1.40E+00 | ± | 1.62E-01 | 7.55E-01 | ± | 2.43E-01 | 2.26E+00 | ± | 4.73E-01 | 1.44E+00 | ± | 5.06E-01 |
| **Glucose MeOX2** | 1.50E-01 | ± | 6.55E-02 | 6.70E-02 | ± | 2.82E-02 | 4.34E-01 | ± | 9.07E-02 | 2.50E-01 | ± | 5.09E-02 |
| **Glycerol** | 3.00E-04 | ± | 0.00E+00 | 3.00E-04 | ± | 0.00E+00 | 3.30E-02 | ± | 3.18E-02 | 1.50E-02 | ± | 2.31E-03 |
| **Glycine** | 3.00E-04 | ± | 0.00E+00 | 8.36E-03 | ± | 6.04E-03 | 2.20E-02 | ± | 7.39E-03 | 1.46E-02 | ± | 1.66E-03 |
| **Isocitrate** | 1.10E-02 | ± | 9.32E-03 | 2.95E-02 | ± | 2.43E-02 | 1.43E-02 | ± | 6.43E-03 | 4.77E-02 | ± | 1.55E-02 |
| **Leucine** | 3.00E-04 | ± | 0.00E+00 | 3.00E-04 | ± | 0.00E+00 | 5.16E-03 | ± | 3.10E-03 | 1.21E-03 | ± | 7.84E-04 |
| **Malate** | 4.49E-01 | ± | 9.42E-02 | 5.76E-01 | ± | 1.44E-01 | 8.69E-01 | ± | 1.22E-01 | 7.09E-01 | ± | 3.99E-02 |
| **Myo-inositol** | 1.48E-01 | ± | 1.05E-01 | 1.07E-01 | ± | 4.61E-02 | 3.54E-01 | ± | 6.42E-02 | 1.92E-01 | ± | 5.53E-02 |
| **Phosphate** | 1.77E-01 | ± | 5.77E-02 | 3.11E-01 | ± | 1.91E-02 | 3.04E-01 | ± | 7.35E-02 | 3.45E-01 | ± | 1.22E-02 |
| **Pyroglutamate** | 1.42E-02 | ± | 1.42E-02 | 3.00E-04 | ± | 0.00E+00 | 5.77E-02 | ± | 9.91E-03 | 3.56E-02 | ± | 3.17E-03 |
| **Quinate** | 2.44E-01 | ± | 8.26E-02 | 3.40E-01 | ± | 7.23E-02 | 3.95E-01 | ± | 1.15E-01 | 4.29E-01 | ± | 6.15E-02 |
| **Serine** | 3.00E-04 | ± | 0.00E+00 | 3.00E-04 | ± | 0.00E+00 | 2.19E-02 | ± | 1.11E-02 | 8.50E-03 | ± | 1.95E-03 |
| **Succinate** | 3.00E-04 | ± | 0.00E+00 | 3.00E-04 | ± | 0.00E+00 | 2.96E-02 | ± | 7.78E-03 | 1.87E-02 | ± | 3.38E-03 |
| **Sucrose** | 9.08E+00 | ± | 3.89E+00 | 9.02E+00 | ± | 1.41E+00 | 1.36E+01 | ± | 4.70E+00 | 9.85E+00 | ± | 2.37E+00 |
| **Threonate** | 1.45E-03 | ± | 1.99E-03 | 3.00E-04 | ± | 0.00E+00 | 3.14E-02 | ± | 7.74E-03 | 1.63E-02 | ± | 5.87E-03 |
| **Threonine** | 3.00E-04 | ± | 0.00E+00 | 3.00E-04 | ± | 0.00E+00 | 8.00E-03 | ± | 1.64E-03 | 2.14E-03 | ± | 2.48E-04 |
| **Valine** | 3.00E-04 | ± | 0.00E+00 | 3.00E-04 | ± | 0.00E+00 | 1.33E-02 | ± | 6.60E-03 | 3.12E-03 | ± | 2.01E-03 |

Legend: Highlighted rows represent metabolites, which were detected in our experiment, with potential roles in plant stress response which were detected in our experiment.

Weights are shown in mg/g fresh weight, zero (0), minimum (3E-04), maximum (53.37), blanks are replaced with 2E-04, detection limit was set at 0.0003.
