# Supplementary material for: Nitrogen limitation and high density responses in rice suggest a role for ethylene under high density stress
Source: BMC Genomics. 2014 Aug 13;15(1):681. doi: 10.1186/1471-2164-15-681 (PMC4138374; doi:10.1186/1471-2164-15-681)
Supplement: Supplementary file 3 — Additional file 3: Growth conditions used in this study are shown in Figure S1. Figure S2. shows an example of MapMan analysis output and demonstrates involvement of ethylene hormone. (DOCX 2 MB) [file 12864_2013_6360_MOESM3_ESM.docx]

**Additional file 3**

Figure S1 Experimental set up of growth conditions used to grow rice under high density (40 plants per bin) versus low density (six plants per bin) and sufficient nitrogen (10mM NO_3_^-^) versus limiting nitrogen (3mM NO_3_^-^).


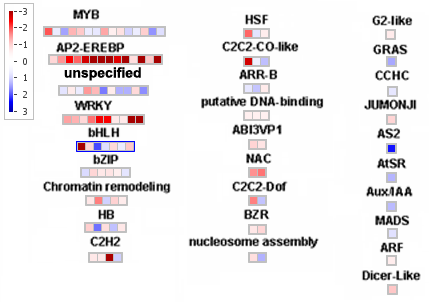


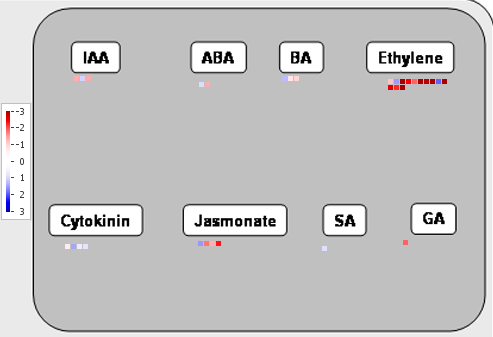


Figure S2 Example of MapMan analysis output comparing differentially expressed genes of plants grown under high versus low density conditions with 2.0 fold change cut off. The genes were assigned into functional categories based on similarities to the genes of Arabidopsis with known function. Each square is representative of an annotated gene according to the MapMan database. The colour of each square corresponds to fold change in expression level in high density responsive transcripts. Ethylene associated genes were over-represented compared to other phytohormones and therefore selected for further analysis.
