# Supplementary material for: Nitrogen limitation and high density responses in rice suggest a role for ethylene under high density stress
Source: BMC Genomics. 2014 Aug 13;15(1):681. doi: 10.1186/1471-2164-15-681 (PMC4138374; doi:10.1186/1471-2164-15-681)
Supplement: Supplementary file 4 — Additional file 4: Summary of AgriGO analysis of differentially expressed genes at 31 days. (DOCX 21 KB) [file 12864_2013_6360_MOESM4_ESM.docx]

Additional file 4: Table S3 AgriGO analysis of differentially (high density vs low density grown plants) responsive genes sorted by biological function in 31 day old rice plants under high density stress

| **GO term** | **Ontology** | **Description** | **# in input list** | **# in BG/Ref** | **p-value** | **FDR** |
| --- | --- | --- | --- | --- | --- | --- |
| GO:0008152 | P | metabolic process | 595 | 19328 | 2.40E-30 | 7.90E-28 |
| GO:0009987 | P | cellular process | 540 | 18214 | 1.30E-19 | 2.10E-17 |
| GO:0009058 | P | biosynthetic process | 275 | 7335 | 1.50E-18 | 1.60E-16 |
| GO:0044238 | P | primary metabolic process | 422 | 13205 | 2.80E-18 | 2.30E-16 |
| GO:0006807 | P | nitrogen compound metabolic process | 214 | 5399 | 2.30E-16 | 1.20E-14 |
| GO:0006139 | P | nucleobase, nucleoside, nucleotide and nucleic acid metabolic process | 214 | 5399 | 2.30E-16 | 1.20E-14 |
| GO:0050896 | P | response to stimulus | 256 | 6928 | 2.90E-16 | 1.40E-14 |
| GO:0044237 | P | cellular metabolic process | 335 | 10280 | 3.10E-14 | 1.30E-12 |
| GO:0006950 | P | response to stress | 179 | 4660 | 2.40E-12 | 8.50E-11 |
| GO:0009628 | P | response to abiotic stimulus | 128 | 3022 | 1.80E-11 | 5.90E-10 |
| GO:0009719 | P | response to endogenous stimulus | 93 | 2015 | 2.60E-10 | 7.80E-09 |
| GO:0032502 | P | developmental process | 134 | 3791 | 3.20E-07 | 8.40E-06 |
| GO:0032501 | P | multicellular organismal process | 129 | 3619 | 3.60E-07 | 8.40E-06 |
| GO:0007275 | P | multicellular organismal development | 127 | 3543 | 3.40E-07 | 8.40E-06 |
| GO:0048856 | P | anatomical structure development | 69 | 1665 | 2.60E-06 | 5.70E-05 |
| GO:0023052 | P | signaling | 90 | 2409 | 4.70E-06 | 9.60E-05 |
| GO:0023046 | P | signaling process | 75 | 1972 | 1.70E-05 | 0.00032 |
| GO:0023060 | P | signal transmission | 75 | 1972 | 1.70E-05 | 0.00032 |
| GO:0007165 | P | signal transduction | 74 | 1951 | 2.20E-05 | 0.00035 |
| GO:0050794 | P | regulation of cellular process | 74 | 1951 | 2.20E-05 | 0.00035 |
| GO:0048869 | P | cellular developmental process | 35 | 710 | 3.40E-05 | 0.00051 |
| GO:0030154 | P | cell differentiation | 35 | 710 | 3.40E-05 | 0.00051 |
| GO:0006629 | P | lipid metabolic process | 56 | 1376 | 3.80E-05 | 0.00054 |
| GO:0009056 | P | catabolic process | 72 | 2007 | 0.00015 | 0.002 |
| GO:0050789 | P | regulation of biological process | 74 | 2132 | 0.00031 | 0.004 |
| GO:0009908 | P | flower development | 30 | 662 | 0.00047 | 0.0059 |
| GO:0048608 | P | reproductive structure development | 30 | 668 | 0.00054 | 0.0063 |
| GO:0003006 | P | reproductive developmental process | 30 | 668 | 0.00054 | 0.0063 |
| GO:0065007 | P | biological regulation | 92 | 2871 | 0.00075 | 0.0085 |
| GO:0022414 | P | reproductive process | 39 | 985 | 0.00092 | 0.01 |
| GO:0006091 | P | generation of precursor metabolites and energy | 23 | 480 | 0.001 | 0.011 |
| GO:0009653 | P | anatomical structure morphogenesis | 43 | 1141 | 0.0013 | 0.013 |
| GO:0009791 | P | post-embryonic development | 66 | 2033 | 0.0031 | 0.031 |
| GO:0040007 | P | growth | 29 | 736 | 0.0042 | 0.041 |
| *GO:0000003* | *P* | *reproduction* | *62* | *1965* | *0.0074* | *0.069* |
| *GO:0009605* | *P* | *response to external stimulus* | *26* | *681* | *0.0094* | *0.085* |
| *GO:0009991* | *P* | *response to extracellular stimulus* | *17* | *393* | *0.011* | *0.099* |
| *GO:0008219* | *P* | *cell death* | *19* | *478* | *0.017* | *0.14* |
| *GO:0016265* | *P* | *death* | *19* | *478* | *0.017* | *0.14* |
| *GO:0015979* | *P* | *photosynthesis* | *14* | *324* | *0.02* | *0.17* |
| *GO:0005975* | *P* | *carbohydrate metabolic process* | *45* | *1439* | *0.023* | *0.19* |
| *GO:0009607* | *P* | *response to biotic stimulus* | *43* | *1404* | *0.035* | *0.27* |
| *GO:0034645* | *P* | *cellular macromolecule biosynthetic process* | *28* | *890* | *0.06* | *0.43* |
| *GO:0044249* | *P* | *cellular biosynthetic process* | *28* | *890* | *0.06* | *0.43* |
| *GO:0009059* | *P* | *macromolecule biosynthetic process* | *28* | *890* | *0.06* | *0.43* |
| *GO:0006412* | *P* | *translation* | *28* | *890* | *0.06* | *0.43* |
| *GO:0007154* | *P* | *cell communication* | *17* | *512* | *0.086* | *0.6* |
| *GO:0051704* | *P* | *multi-organism process* | *12* | *337* | *0.093* | *0.62* |
| *GO:0009856* | *P* | *pollination* | *12* | *337* | *0.093* | *0.62* |
| *GO:0016049* | *P* | *cell growth* | *17* | *567* | *0.16* | *0.99* |
| *GO:0090066* | *P* | *regulation of anatomical structure size* | *17* | *567* | *0.16* | *0.99* |
| *GO:0008361* | *P* | *regulation of cell size* | *17* | *567* | *0.16* | *0.99* |
| *GO:0032535* | *P* | *regulation of cellular component size* | *17* | *567* | *0.16* | *0.99* |
| *GO:0043412* | *P* | *macromolecule modification* | *82* | *3977* | *0.84* | *1* |
| *GO:0009790* | *P* | *embryonic development* | *23* | *810* | *0.17* | *1* |
| *GO:0044267* | *P* | *cellular protein metabolic process* | *110* | *4849* | *0.53* | *1* |
| *GO:0044260* | *P* | *cellular macromolecule metabolic process* | *115* | *5364* | *0.77* | *1* |
| *GO:0016043* | *P* | *cellular component organization* | *48* | *1935* | *0.29* | *1* |
| *GO:0010467* | *P* | *gene expression* | *28* | *1076* | *0.27* | *1* |
| *GO:0065008* | *P* | *regulation of biological quality* | *19* | *863* | *0.59* | *1* |
| *GO:0006810* | *P* | *transport* | *75* | *3278* | *0.5* | *1* |
| *GO:0006464* | *P* | *protein modification process* | *82* | *3977* | *0.84* | *1* |
| *GO:0051234* | *P* | *establishment of localization* | *75* | *3278* | *0.5* | *1* |
| *GO:0051179* | *P* | *localization* | *75* | *3278* | *0.5* | *1* |
| *GO:0019748* | *P* | *secondary metabolic process* | *14* | *583* | *0.46* | *1* |
| *GO:0019538* | *P* | *protein metabolic process* | *139* | *6088* | *0.5* | *1* |
| *GO:0043170* | *P* | *macromolecule metabolic process* | *144* | *6691* | *0.79* | *1* |
| *GO:0006259* | *P* | *DNA metabolic process* | *6* | *589* | *0.99* | *1* |

Note: Italicized GO terms are not enriched according to false discovery rate cut-off of 0.05
