# Supplementary material for: Nitrogen limitation and high density responses in rice suggest a role for ethylene under high density stress
Source: BMC Genomics. 2014 Aug 13;15(1):681. doi: 10.1186/1471-2164-15-681 (PMC4138374; doi:10.1186/1471-2164-15-681)

Additional file 6

## Cluster analysis of differentially expressed entities.

### Introduction

The data consist of measurements on each gene at two different time points (where time point A represents 21 days and B 31 days), two plant densities (high density and low density) as well as two different nitrogen conditions (high nitrogen and low nitrogen concentration). This resulted in eight different measurements per gene. For our analysis, each individual time point was analyzed separately resulting in a 4-dimensional data set for each time point. A two-step clustering strategy was used for the analysis of each time point.

Step 1: Using the measurements on all 4 conditions, the genes were clustered via a model-based clustering approach for each time point separately. This step ensures genes that follow a similar trend are clustered together. Model-based clustering assumes that the population is a convex combination of probability densities; i.e., that a mixture of subpopulations each with its own mean and covariance structure. In the analysis, Gaussian parsimonious clustering models (GPCM; Celeux and Govaert, 1995) was used, a subset of which is implemented in the R package mclust (Fraley et al., 2012). This family of model utilizes eigen-decomposed component covariance structures.

Step 2: The measurements on the four conditions from each component were split according to the plant density. This resulted in two-dimensional data such that the first set contained expression levels under LN and HN, and HD and the second set contained expression levels under LN and HN, and LD. Each component was analyzed using k-means clustering (Hartigan and Wong, 1979) with k = 2; k-means clustering partitions the data into k clusters such that each observation belongs to the cluster with the nearest mean. If there is a change in expression levels at either high or low nitrogen conditions between low and high densities, the resulting clusters will represent the expression levels at low and high densities. In our case, we are using k=2 and hence the observations are partitioned into two clusters. The black and dotted lines represent the mean expression of those two k-mean clusters (i.e if black line represents the mean expression of k-means cluster number 1 then dotted line will represent the mean expression of k-mean cluster number 2).

### Analysis

#### Pre-processing

The initial step of the analysis was to lter out the genes that show no differential expression across varying conditions. Because our primary focus is on identifying genes that show differential expression under different densities, genes that showed three fold change under high and low density while keeping the nitrogen constant were selected. Out of 21,179 genes, 426 genes showed differential expression at time point A and 707 genes showed differential expression at time point B.

#### Analysis of time point A

The first step of the analysis was to cluster genes using expression levels of all 4 conditions (AHH, AHL, ALH and ALL) using mclust. The abbreviation AHH stands for expression level at time point A, high density and high nitrogen. Similarly, the abbreviation AHL stands for expression level at time point A, high density and low nitrogen. The algorithm was run for 1; : : : ; 10 groups and a mixture of multivariate normal distributions with 8 components (groups) as seen in Figure 3A. For the second step, the genes in each component was then split based on density so that AHH and AHL were in one set and ALH and ALL expression levels were in another. The resulting data was then analyzed using k-means with k = 2 for all 8 components individually. Figure S4 shows the expression levels from high to low nitrogen for both high and low density and the black line shows the estimated expression level. When the expression of a gene at low or high nitrogen conditions differs among low and high densities, the estimated clusters correspond to the different expression levels at low and high densities. However, when there is no difference in expression of genes at low or high nitrogen conditions among low and high densities, the estimated clusters do not correspond to low and high densities. All the genes in clusters 1, 6 and 7 show different mean expressions for high or low nitrogen conditions under low and high densities. Clusters 5 and 8 also seem to have a difference in expression levels under low and high densities (with very few genes as an exception). Clusters 2 and 4 have a high number of misclassifications and the genes in cluster 3 show no change in the expression trend under low and high densities. The list of genes in each cluster is provided in a separate file.

Again, the first step of the analysis was to cluster genes using expression levels at all 4 conditions (BHH, BHL, BLH and BLL) using mixtures of multivariate normal distributions with eigen-decomposed covariance e structure. This was implemented using the R package mclust. The algorithm was run for 1; : : : ; 10 groups and resulted in the selection of multivariate normal distribution with 7 components (groups) as seen in Figure 3B. For the second step, the genes in each component were then split according to density so that BHH and BHL were in one set and BLH and BLL expression levels were in another. The resulting data were then analyzed using k-means with k = 2 for all 7 components individually. Figure S5 shows the expression levels from high to low nitrogen in grey for both high and low density and the black line shows the estimated expression levels. All the genes in clusters 3, 4 and 7 show different mean expression for high or low nitrogen conditions under low and high densities. Cluster 5 also seems to have a difference in expression levels under low and high densities (with a couple of genes as exception). Clusters 1 and 2 have a high number of misclassifications and the genes in cluster 6 show no change in expression trend under low and high densities. The list of genes in each cluster is provided in a separate file.

Comments

The data was also analyzed using other clustering approaches. Parsimonious Gaussian mixture models (PGMM; McNicholas and Murphy, 2008) for step 1 and k-means with k = 2 for step 2. The PGMM utilizes a latent factor structure for the data and is available in the R package pgmm (McNicholas et al., 2011). mclust for step 1 followed by mclust with G = 2 components for step 2. pgmm for step 1 followed by mclust with G = 2 components for step 2. All these approaches gave similar results. This ensures that the results are not just obtained by chance and the clustering approaches are in agreement with one another.


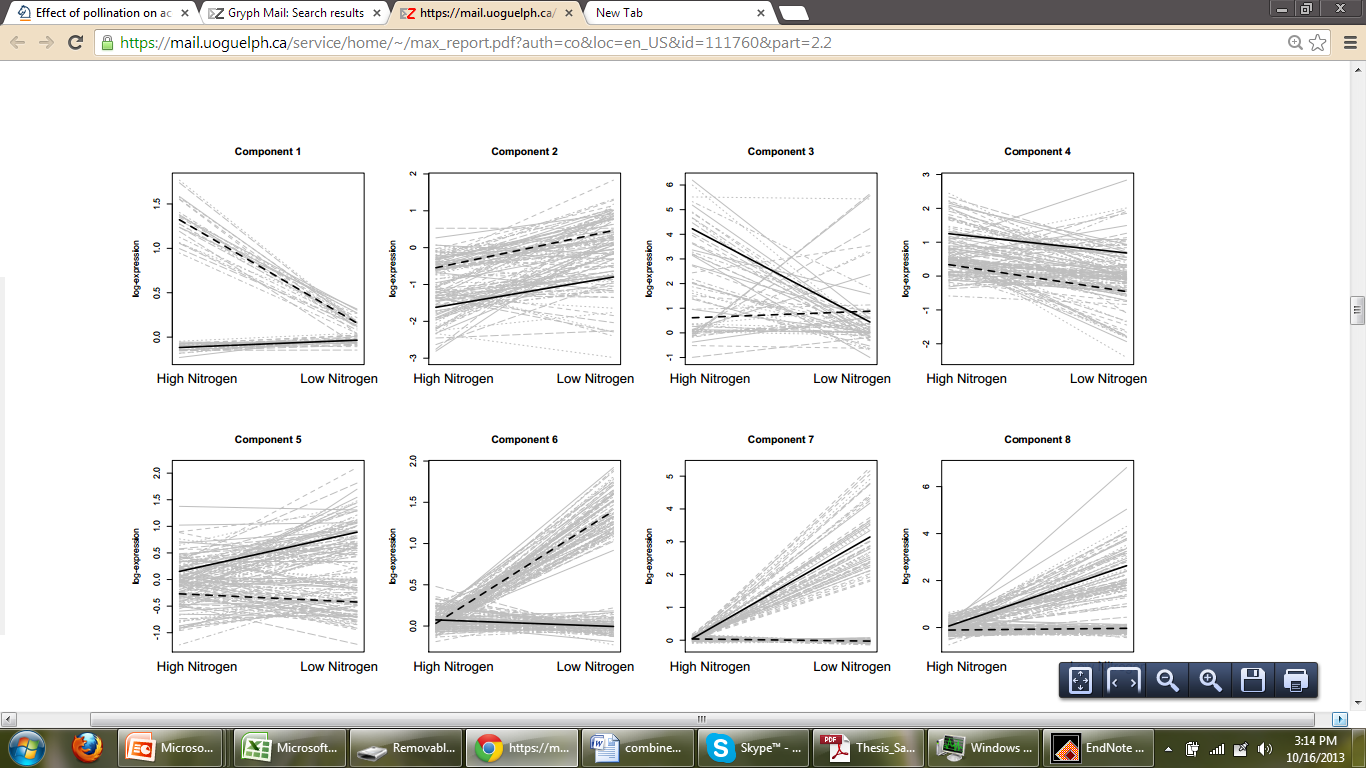


Figure S4 The mean expression of the partitioning of each cluster using k-means with k = 2 in black and the expression profiles of each gene under high and low nitrogen conditions at both low and high densities for time point A.

Table S6 Clustering results of step 2 using k-means with k = 2 for all components of time point A.


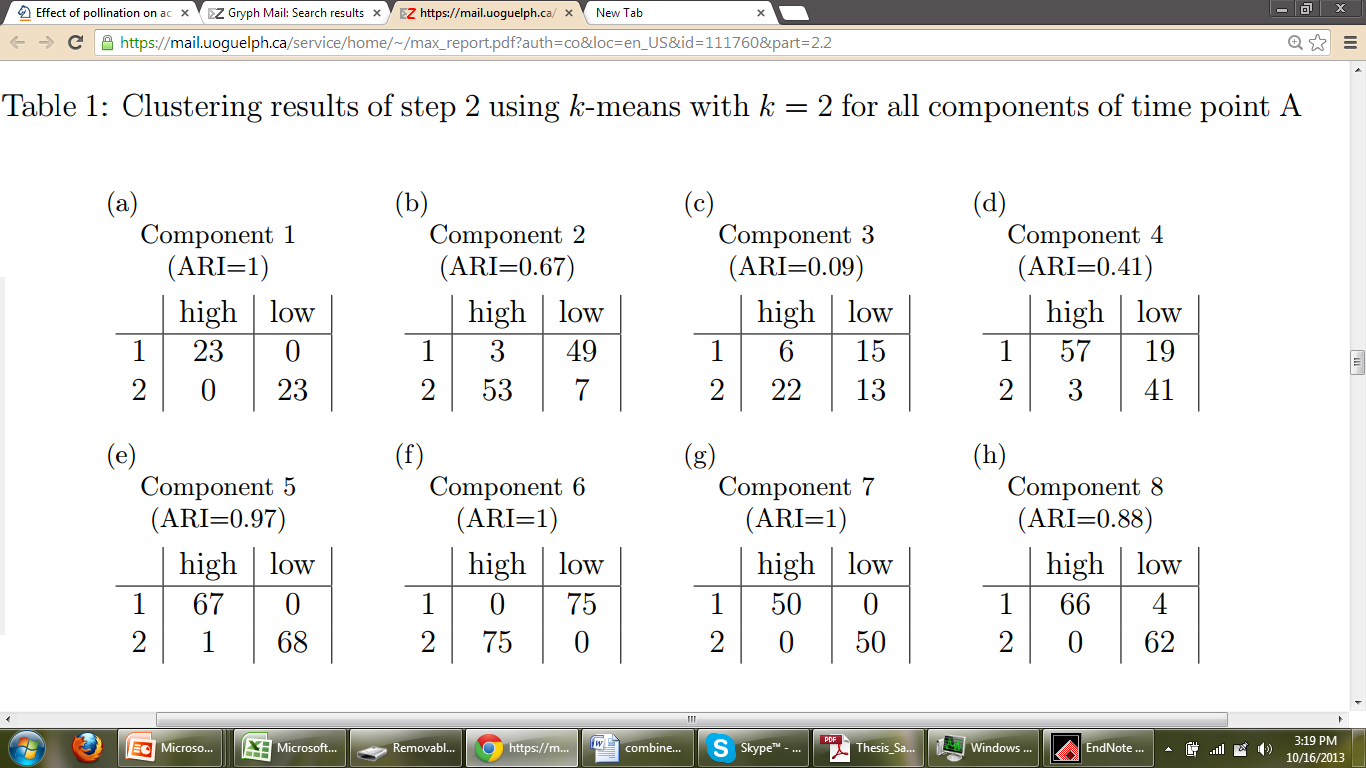


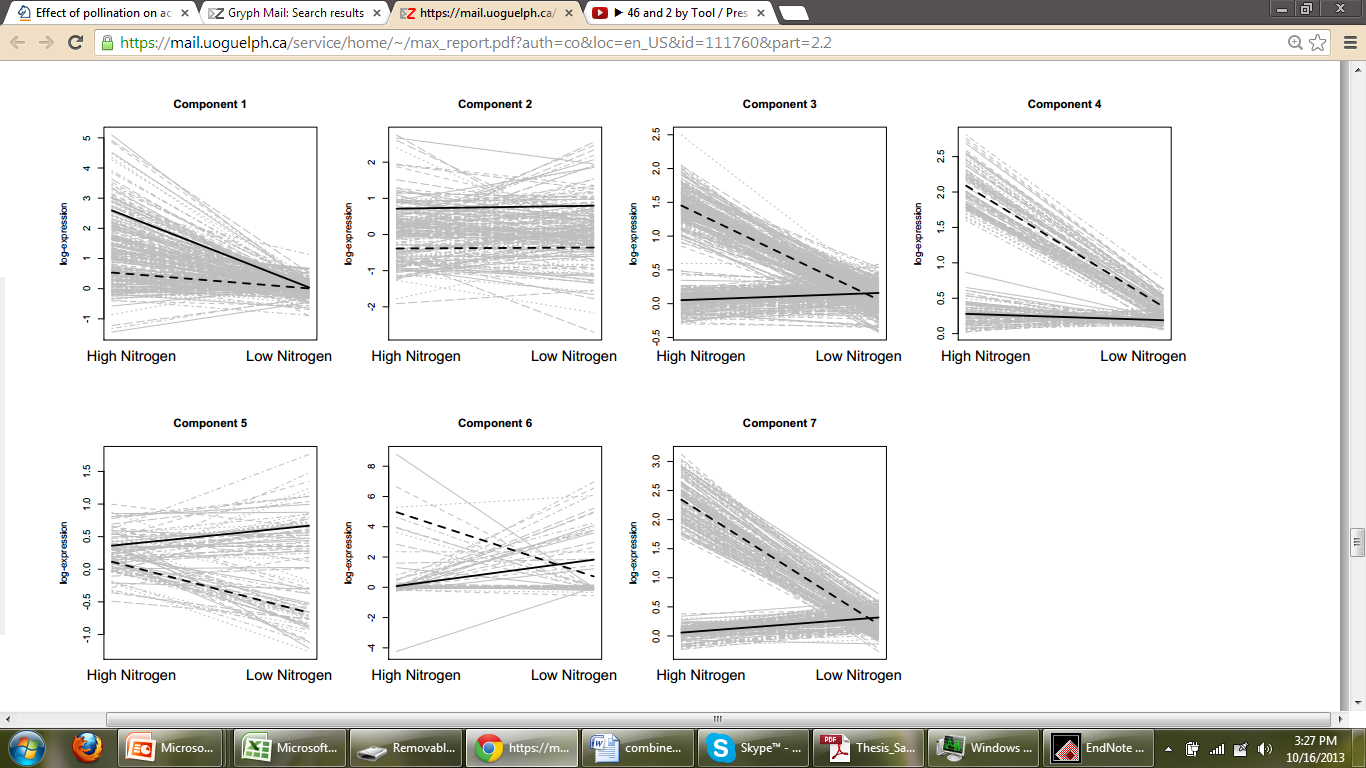


Figure S5 The mean expression of the partitioning of each cluster using k-means with k = 2 in black and the expression profiles of each gene under high and low nitrogen condition at both low and high densities for time point B.

Table S7 Clustering result of step 2 using k-means with k = 2 for all component of time point B.


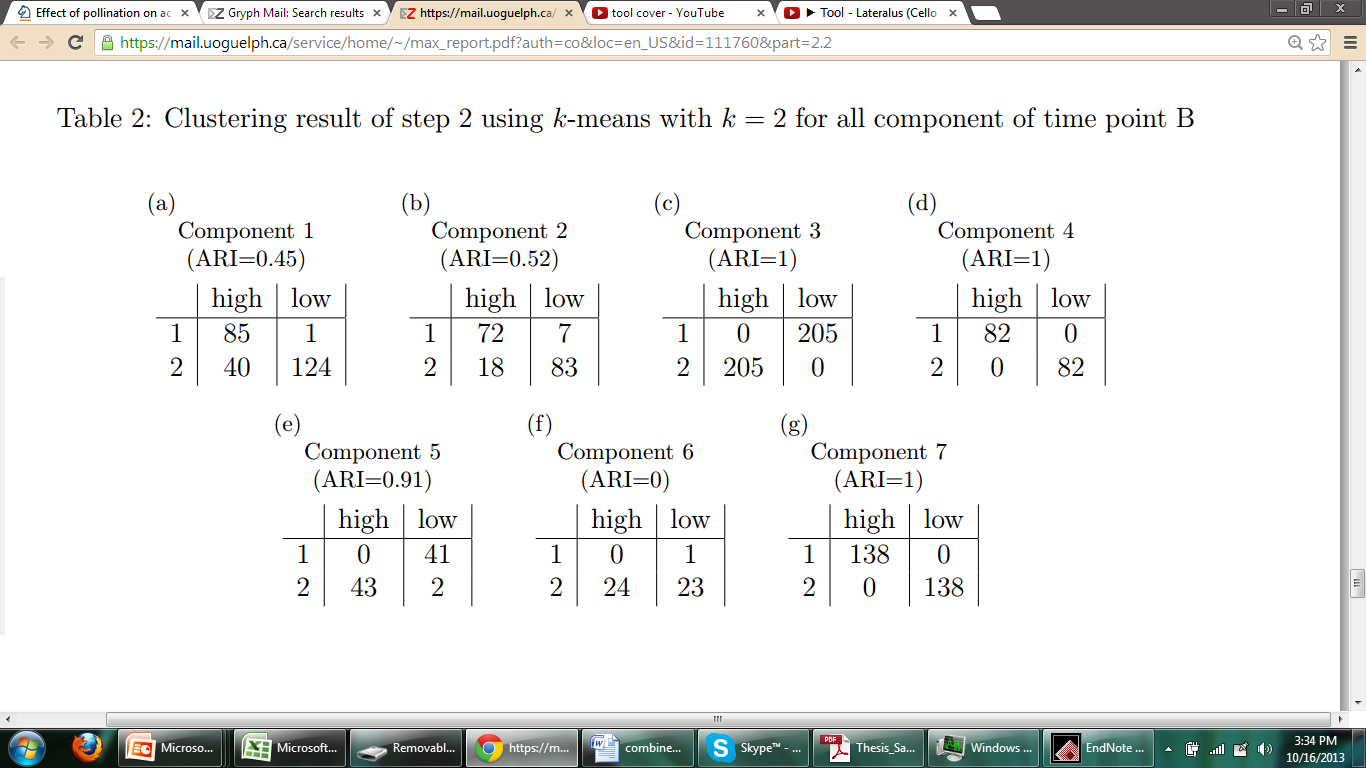

Supplement: Supplementary file 6 — Additional file 6: Detailed description of cluster analysis. (DOCX 943 KB) [file 12864_2013_6360_MOESM6_ESM.docx]
